# Endocytosis of Chikungunya Virus into Mammalian Cells: Role of Clathrin and Early Endosomal Compartments

**DOI:** 10.1371/journal.pone.0011479

**Published:** 2010-07-08

**Authors:** Eric Bernard, Maxime Solignat, Bernard Gay, Nathalie Chazal, Stephen Higgs, Christian Devaux, Laurence Briant

**Affiliations:** 1 Centre d'études d'agents Pathogènes et Biotechnologies pour la Santé (CPBS), CNRS-UMR5236, Université Montpellier 1,2, Montpellier, France; 2 Department of Pathology and Center for Biodefense and Emerging Infectious Diseases, University of Texas Medical Branch, Galveston, Texas, United States of America; Institut Pasteur, France

## Abstract

**Background:**

The replicative cycle of chikungunya virus (CHIKV), an alphavirus that recently re-emerged in India and in Indian Ocean area, remains mostly unknown. The aim of the present study was to investigate the intracellular trafficking pathway(s) hijacked by CHIKV to enter mammalian cells.

**Methodology/Principal Findings:**

Entry pathways were investigated using a variety of pharmacological inhibitors or overexpression of dominant negative forms of proteins perturbating cellular endocytosis. We found that CHIKV infection of HEK293T mammalian cells is independent of clathrin heavy chain and- dependent of functional Eps15, and requires integrity of Rab5-, but not Rab7-positive endosomal compartment. Cytoskeleton integrity is crucial as cytochalasin D and nocodazole significantly reduced infection of the cells. Finally, both methyl β-cyclodextrin and lysomotropic agents impaired CHIKV infection, supporting that a cholesterol-, pH-dependent step is required to achieve productive infection. Interestingly, differential sensitivity to lysomotropic agents was observed between the prototypal 37997 African strain of CHIKV and the LR-OPY1 virus isolated from the recent outbreak in Reunion Island.

**Conclusions:**

Together our data indicate that CHIKV entry in its target cells is essentially mediated by clathrin-independent, Eps15-dependent endocytosis. Despite that this property is shared by the prototypal 37997 African strain of CHIKV and the LR-OPY1 virus isolated from the recent outbreak in La Réunion Island, differential sensitivity to lysomotropic agents may support that the LR-OPY1 strain has acquired specific entry mechanisms.

## Introduction

Chikungunya virus (CHIKV) is member of the Togaviridae family. It belongs to the Semliki Forest Virus (SFV) group and is closely related to other viruses from the family of arthrogenic alphaviruses, with a marked similarity with o'nyong-nyong virus [1]. CHIKV is an enveloped single-stranded positive RNA virus. The coding sequence comprising the nonstructural and structural polyproteins is of approximately 12 kb [2]. According to the genomic organization of other alphaviruses, the genome of CHIKV is considered to be: 5′-nsP1-nsP2-nsP3-nsP4-junction region-C-E3-E2-6K-E1-poly(A)-3′. Chikungunya virions are approximately 70 nm in diameter [3], [4] and contain a spherical capsid with icosahedral symmetry. Viral glycoproteins E1 and E2, embedded in the lipid bilayer surrounding the viral capsid, direct the virus attachment to the host cell and the fusion with cellular membranes.

Chikungunya virus is transmitted to humans by mosquito vectors during blood feeding. Since the first isolation of CHIKV more than 50 years ago in Eastern and Central Africa [5], chikungunya epidemics have been repeatedly recorded from various countries in central, southern and western Africa [6], [7], [8], with a recent episode traced in Kenya [9]. In Asia, CHIKV was isolated from Bangkok, Thailand in 1958 [10] and repeated outbreaks have been recorded since early 1960s [11], [12], [13], [14]. From February 2005 to June 2006, CHIKV caused an explosive burst in the Indian Ocean, originally documented for Reunion Island [15] and next in neighboring islands Seychelles, Madagascar, Mauritius and Mayotte [16], [17]. During the same period, an outbreak of unprecedented magnitude started in India, where more than 1.4 million persons have been infected [18], [19]. The detection of chikungunya fever cases in travellers returning from known outbreak areas to regions where mosquito species competent for CHIKV are widely distributed [20], [21], [22], together with the CHIKV epidemics detected during summer 2007 in the Emilia-Romagna Region, central Italy [23], indicates that CHIKV may become a general threat for human health in temperate areas.

Following CHIKV exposure, infected individuals display symptoms of incapacitating polyarthralgia with join pains, severe myalgia, and a cutaneous rash. Despite chikungunya infection is often a self-limiting illness and most patients recover completely over a few weeks, 5% to 10% of patients experienced chronic symptoms lasting a year or more [24], [25]. During the recent outbreak in Reunion Island, the detailed epidemiologic survey revealed the appearance of severe neurologic symptom. Moreover, at the end of the epidemic, an estimated 255 fatalities were attributed to chikungunya infection [15]. Such symptoms have never been documented in chikungunya epidemics raging in other parts of the world. Although most countries where Chikungunya rages lack of appropriate surveillance system, a special virulence of the virus circulating during La Reunion outbreak can be questioned. Phylogenetic analysis performed at different time points during the epidemic revealed that genomes circulating in the Indian Ocean were derived from an African ancestor belonging to the Eastern-Central-South African (ECSA) group of viruses [26]. Interestingly, the presence of the A226V mutation in the E1 envelope gene sequence was detected in genomes of isolates present at the epidemic peak [26]. During the same period, the A226V mutation was evidenced in genomes of highly pathogenic viruses circulating in the Indian state of Kerala [27] indicating that this viral subtype retains a selective advantage. Substitution of the A226 residue in E1, that was previously observed to impact membrane fusion and more precisely cholesterol dependence, using a SFV model of infection [28], [29], was proposed to be responsible for the increased transmissibility and higher epidemic potential of CHIKV [26]. Although the role of this mutation is unknown for human pathogenesis, it facilitates viral dissemination in *Aedes albopictus* mosquito that served as a vector for CHIKV during the recent Indian and the Indian Ocean area epidemics [30], [31].

If the replication cycle of alphaviruses has been largely studied [2] and differences have been reported regarding the mechanisms used to enter their target cell [32], [33], [34], CHIKV host-cell interactions remain poorly documented [35]. In light of the emergence of CHIKV in previously unaffected areas, and of the recent report of a considerable proportion of severe symptomatic cases, a thorough understanding of the routes used by this viral agent to infect its target cells will help to rationally design antiviral strategies that will protect humans against infection with CHIKV, and perhaps related viruses with potential emergence in temperate areas. In the present study, we performed a detailed investigation of the largely unknown mechanisms of cellular entry of CHIKV into mammalian cells. We examined the entry of CHIKV into epithelial HEK293T cell cultures using a variety of strategies perturbating cellular endocytosis, including treatment with chemical inhibitors, expression of dominant negative proteins or RNA interference. Given that a higher epidemic potential was associated with the E1-A226V mutation, we performed comparative studies using the 37997 African prototype strain and the LR-OPY1 virus isolated during Reunion Island epidemic that bears a mutated envelope glycoprotein. We demonstrate here that CHIKV preferentially uses a clathrin-independent, Eps15-dependent pathway to enter mammalian epithelial cells. Moreover, functional Rab5-, but not Rab7-dependent compartments are required for CHIKV to infect these cells. We also show that membrane cholesterol as well as endosomal pH acidification is required to reach a productive infection. Finally, integrity of the cell cytoskeleton is required. Disregarding the presence of the A226V mutation in E1 gene, similar requirement for entry were shared by the 37997 strain and the LR-OPY1 isolate, including cholesterol dependence. However, some differences observed regarding sensitivity of these strains to lysomotropic agents suggest that the LR-OPY1 strain may have acquired specific entry mechanisms. Altogether, our data describe the cellular pathway used by CHIKV to enter mammalian epithelial cells.

## Methods

### Cell lines

Human embryonic kidney (HEK293T) cells [36] were maintained at 37°C under 5% CO_2_ in DMEM (Lonza) containing 10% inactivated fetal calf serum and 1% antibiotics. BHK-21 and Vero cells used for virus production and titration were cultured under similar conditions.

### Production of viral stocks and titration

The CHIKV subgenomic clones pCHIKic4 (37997 strain) and pCHIK-LRic (LR-OPY1 strain), and the full length CHIKV subgenomic clones expressing GFP, pCHIKic-3′GFP (37997-GFP strain) and pCHIKic-LR-3′GFP (LR-OPY1-GFP strain) have been previously described [30], [37]. Each infectious clone was transcribed *in vitro* from the SP6 promoter using the mMESSAGE mMACHINE kit (Ambion) following manufacturer's instructions. RNA (0.5 µg) was then electroporated into 5×10^6^ BHK-21 cells with two pulses at 1.5 kV, 25 µF and ∞Ω. After two days, cell culture supernatant was harvested, filtered through 0.22 µm filters, aliquoted and stored at −80°C. Viral stocks were tittered using Vero cell plaque assays [38].

### RNA interference

Subconfluent cultures of HEK293T in 24-well plates were transfected with 1.2 pmol of synthetic double stranded siRNA complexes (Ambion) and 2 µl Interferin (PolyPlus Transfection), in a final volume of 600 µl serum-free medium. The clathrin heavy chain target sequence was GGUUGCUCUUGUUACGGAU. The On Target plus control pool siRNA (Dharmacon) was used as a control. After 72 h in culture, an aliquot of the cells was assayed for clathrin heavy chain expression by immunoblotting using the (N-19) anti-clathrin heavy chain mAbs from Santa Cruz Biotechnologies Inc. The remaining cells were challenged with CHIKV strains used at a multiplicity of infection (m.o.i.) of 5.

### Inhibition of endosomal acidification

Inhibition of endosomal acidification was reached through the use of chloroquine (100 µM), monensin (1 µM), NH_4_Cl (20 mM) or bafilomycin A1 (100 nM). Control cells were treated with equivalent dilutions of solvent. HEK293T cells (8×10^4^) were seeded into 24 well plates, 24 hours before drug treatment. Cells were then incubated for 1 h in fresh medium in the presence of inhibitor before exposure to CHIKV at a m.o.i. of 5. After 2 hours of infection, the cells were extensively washed with DMEM and cultured for another 16 h in the presence of fresh medium before analysis. To evaluate the capacity of drugs to inhibit intracellular steps of viral replication, cells were challenged with CHIKV (m.o.i. of 5), washed and maintained in culture for 4 hours before the drugs or equivalent concentration of solvent are added to culture medium. Infection levels were monitored after an additional 16 h in culture.

### Cholesterol depletion

Cholesterol depletion was achieved by treatment of HEK293T cells with methyl β-cyclodextrin (Sigma). HEK293T cells (3×10^5^) were seeded into 6-wells plates. After 24 h, cells were washed twice with medium containing 10% FCS and treated with methyl β-cyclodextrin for 1 h at 37°C in serum-free DMEM. After washing with cold PBS, cells were chilled for 10 min on ice to prevent endocytosis before addition of CHIKV (m.o.i. of 5). Following incubation for 2 h at 37°C, the drug was removed and the infection allowed to proceed for 16 h at 37°C before analysis.

### Cytoskeleton disruption

Nocodazole or cytochalasin D, purchased from Sigma, were added to HEK293T cells at various concentrations ranging from 10 µM to 50 µM. After 2 hours in culture, the medium was removed and the cells were challenged with CHIKV (m.o.i. of 5) in the presence of the drug. CHIKV replication was monitored after an additional 16 h in culture.

### Acid-mediated endocytosis by-pass assay

Cells were pre-treated with inhibitors at their optimal concentration for the appropriate time. Then the virus (m.o.i. of 5) was allowed to bind to the cells for 30 min at 4°C. Infection was allowed to proceed by incubating the cells for 30 min at 37°C either in neutral (pH 7.4) or acid (pH 5.0) PBS. After washes with neutral PBS, the cells were cultured in DMEM medium for 16 hours in the presence of the inhibitor. Infection of the cells was monitored by flow cytometry.

### Inhibition of cells endocytosis

Inhibition of endocytosis was achieved by transfection of the pEGFP-Eps15-PEΔ95/295 plasmid encoding a GFP-tagged dominant negative (DN) form of the epidermal growth factor receptor pathway substrate 15 (Eps15). The pEGFP-Eps15-WT encoding GFP-tagged wild type (WT) Eps15 was used as a control. Expression of WT or DN Rab5 and Rab7 GTPases was obtained by transfection of pQCXIP EGFP-Rab5-WT or pQCXIP EGFP-Rab5-S34N and pQCXIP EGFP-Rab7-WT or pQCXIP EGFP-Rab7-T22N plasmids respectively. Plasmids (1 µg) were transfected in semi-confluent HEK293T cells by calcium phosphate precipitation. After twenty-four hours, culture supernatant was removed and replaced by fresh medium. Then, the cells were challenged with CHIKV and analyzed for virus susceptibility using either immunofluorescence experiments or flow cytometry assays.

### Indirect immunofluorescence (IF) and confocal microscopy

Transfected cells challenged with CHIKV (m.o.i. of 5) were washed in PBS, fixed and permeabilized for 30 minutes at 37°C in a solution containing 4% paraformaldehyde and 0.1% Triton X100 (Sigma). The cells were saturated with PBS containing 2% FCS, washed twice in PBS and incubated for 1 h at room temperature with mAbs C42, an IgG2a antibody raised to Semliki Forest nucleocapsid that reacts with CHIKV capsid protein, [39] kindly provided by Dr. Irene Greiser-Wilke, School of Veterinary Medicine (Hannover, Germany). After washes in PBS, cells were incubated with Alexa Fluor 594-conjugated anti-mouse Ig secondary antibody for 30 minutes at RT (Invitrogen). Coverslips were washed, mounted with ProLong Gold antifade reagent (Invitrogen) and examined using a confocal microscope. Transferrin uptake control was performed by incubating the cells with Alexa-594-labelled transferrin (5 µg/ml) for 30 min at 37°C. Cells were then washed to remove any uninternalized ligand, fixed and processed as described above.

### Flow Cytometry

Cells challenged for 16 h with CHIKV (m.o.i. of 1) were fixed with a 4% paraformaldehyde solution for 20 min at 4°C. Intracellular labelling of CHIKV antigens was performed by incubating the cells for 1 h at 4°C with mAbs C42 in the presence of 0.1% saponine. After washes with 0.1% saponine in PBS, bound antibodies were detected by addition of Spectral Red (SPRD) conjugated secondary IgG (Beckman Coulter) and incubation for 45 min at 4°C. SPRD IgG isotype was used as a control. Fluorescence intensity was recorded (20,000 events) on a COULTER EPICS XL Flow Cytometer (Beckman Coulter).

## Results

### CHIKV infection of mammalian cells is dependent on Eps15

**Figure 1:**
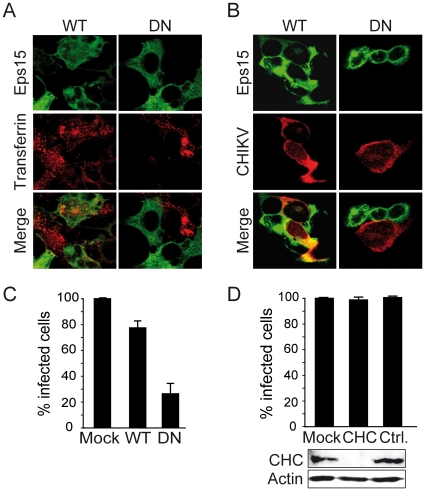
Functional Eps15 but not clathrin heavy chain expression is required for infection by CHIKV. HEK293T cells were transfected with plasmids encoding either a GFP-labelled wild type Eps15 (WT) or the GFP-tagged dominant negative Eps15Δ95/295 mutant (DN) and maintained for 36 h in culture. (A) Cells were incubated with Alexa595-labelled transferrin for 30 min at 37°C and uptake by transfected cells was monitored using immunofluorescence microscopy. (B) Transfected cells were challenged with CHIKV for 4 h at 37°C (37997-CHIKV strain used at a m.o.i. of 5) and the presence of intracellular viral antigens was detected using a mAbs C42 reacting with CHIKV and Texas-red-conjugated secondary antibodies. (C) Tansgene-expressing cells positive for intracellular CHIKV antigens were quantified from panel (B) by flow cytometry. Values are the mean of 4 separate experiments performed in triplicate ± SD. (D) Cells transfected with siRNA targeting the clathrin heavy chain (CHC) or with control siRNA (Ctrl.) were challenged with 37997-CHIKV. Levels of infection were monitored by quantification of GFP-positive cells using flow cytometry (upper panel). Levels of CHC expression in transfected cells were analyzed by immunoblot analysis. Actin expression was used to monitor proteins level in each sample (lower panel).

To characterize the entry route used by CHIKV, we examined the effects on CHIKV infection of a series of chemical inhibitors or well-characterized dominant negative (DN) mutants or siRNA interfering with endocytosis. The experimental model used to achieve this goal consisted of *in vitro* infection of the mammalian epithelial-derived HEK293T cell line, previously reported to be highly susceptible to CHIKV [40]. Considering this experimental model we addressed the role of Eps15, an actor of early endocytosis. Eps15 is a component of clathrin-coated pits where it interacts with adaptor protein (AP)-2, the major clathrin adaptor complex. More recently, Eps15 was also reported to play an important function in coupling cargo internalization to clathrin-independent endocytosis [41]. The role of Eps15 in mediating entry of CHIKV into mammalian cells was addressed by mean of overexpression of Eps15Δ95/295, a dominant negative (DN) mutant of Eps15 [42]. Transfection of GFP-tagged wild-type Eps15 (WT Eps15) or Eps15Δ95/295 (DN Eps15) resulted in over 85% GFP expression as determined by flow cytometry analysis (data not shown). To confirm the effect of Eps15-encoding vectors on endocytosis, uptake of transferrin, a ligand known to enter into the cells using an Eps15-dependent pathway, was investigated (Figure 1A). As expected, transferrin endocytosis was abrogated in cells expressing DN Eps15 but remained unaffected in cells expressing WT Eps15. When transfected cells were challenged with the 37997 CHIKV strain, the percentage of cells positive for viral antigens was markedly reduced in cells expressing DN Eps15 when compared with that in cells over-expressing WT Eps15 (Figure 1B). To quantitatively assess the contribution of functional Eps15 in CHIKV infection, similar experiments were performed where cells expressing WT Eps15 or DN Eps15, exposed to CHIKV, were permeabilized and labelled for intracytoplasmic viral antigens using antibodies reacting with the viral capsid protein before flow cytometry analysis. For each condition, the percentage of CHIKV-infected cells was monitored among the population of transgene-expressing cells (GFP expressing cells). A 60% reduction of CHIKV-positive cells resulted from disruption of Eps15 activity following expression of DN Eps15 when compared with mock transfected cells (Figure 1C). In contrast, overexpression of WT Eps15 did not significantly alter the level of CHIKV infection when compared with control conditions. Altogether, our results indicate that CHIKV entry into HEK293T cells requires functional Eps15.

### Evaluating the role of clathrin-dependent endocytosis in CHIKV entry into epithelial mammalian cells

To decipher with the role of clathrin-dependent endocytosis in CHIKV infection, we investigated the consequence of clathrin heavy chain (CHC) knock out using RNA interference. At 72 h post-transfection, we found that siRNA specific for CHC almost abolished clathrin expression whereas protein levels remained unaffected in cells transfected with irrelevant siRNA (Figure 1D, lower panel). Then, siRNA-transfected cells were challenged with CHIKV. Using this experimental model, no significant difference was observed between cells knocked-down for CHC or cells expressing control siRNA (Figure 1D, upper panel). Accordingly, inhibition of CHC expression does not modify the capacity of HEK293T cells to be infected with CHIKV, supporting that the pathways used to infected this particular cell line is independent of endocytosis by clathrin-coated pits. Because of this unexpected result, we have reanalyzed the role of clathrin-dependent endocytosis using a second epithelial cell line. Using experimental procedures identical to those used for HEK293T cells, we found that CHIKV infection level was similar in Hela cells expressing control siRNA or showing a drastic decrease in CHC expression due to transfection of specific RNA (Figure S1). These results corroborate data obtained with HEK293T cells.

### CHIKV infection of mammalian cells is inhibited by lysomotropic agents

**Figure 2:**
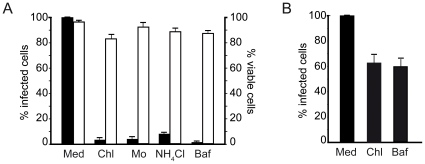
CHIKV infection of HEK293T cells is inhibited by lysomotropic agents. (A) Cells were incubated in medium supplemented with drug solvent (Med) or medium supplemented with appropriate concentrations of lysomotropic agents inhibiting endosomal acidification: chloroquine (Chl), monensine (Mo), amonium chloride (NH_4_Cl), bafilomycine A1 (Baf). The cells were challenged with the 37997-GFP CHIKV strain used at a m.o.i. of 5. The percentage of GFP-expressing cells was determined by flow cytometry analysis (black bars). For each experiment, the number of viable cells was determined after exclusion coloration with Trypan Blue (white bars). Values are expressed as a percentage of untreated controls. Each value is the mean of 3 separate experiments performed in triplicate ± SD. (B) Appropriate concentrations of lysomotropic drugs were added to CHIKV infected cells 4 hours after viral challenge (m.o.i. of 5). After an additional 16 h in culture, percentage of infected cells was determined by flow cytometry.

The contribution of low endosomal pH was next investigated in CHIKV infection. HEK293T cells were treated with various lysomotropic agents prior to viral challenge, including primary amines ammonium chloride (NH_4_Cl), chloroquine that crosses the membranes and buffers the endosomal medium against acidification, monensin, a cationic ionophore which uncouples the sodium/potassium gradient across endosomal membranes required for proton accumulation into endosomes, and bafilomycin A1, a mammalian vacuolar-type-H^+^-ATPase inhibitor that prevents acidification of the endosomal compartment. Then the cells were challenged with the well characterized 37997-GFP CHIKV clone containing a green fluorescent protein (GFP) coding sequence in the 3′of the open reading frame coding for the non structural proteins [37]. We previously reported the relevance of the 37997-GFP subgenomic clone for molecular studies of CHIKV life cycle [37]. Viral inputs required to reach significant, non saturating levels of infection in this particular cell line (fixed to GFP expression in 60% of the cell culture) were established from preliminary experiments (Figure S2). After 16 h in culture, quantification of GFP-positive cells by flow cytometry was used to determine susceptibility to CHIKV. Addition of 100 µM chloroquine prior to virus exposure dramatically reduced CHIKV-driven GFP expression without affecting cell viability (Figure 2A). Similar range of inhibition was measured using a lower dose of chloroquine (10 µM) (data not shown). Comparable results were obtained when the cells were exposed to monensin or NH_4_Cl or bafilomycin A1 before addition of CHIKV to the cell culture. In order to ascertain that inhibition of CHIKV infection was not due to any effect of inhibitors on a post-fusion step of the virus life cycle, similar experiments were repeated where infection was allowed to proceed for 4 hours before drug treatment. In these conditions, the presence of 100 µM chloroquine or 100 nM bafilomycine allowed infection of over 60% of cells when compared with control conditions (Figure 2B). These data support that both inhibitors have a limited effect on post-fusion steps of CHIKV replication. Thus early CHIKV infection is sensitive to endosomal pH neutralization and requires acidification of the endosomal compartment to productively infected mammalian epithelial cells.

### CHIKV infection is inhibited by methyl β-cyclodextrin treatment

**Figure 3:**
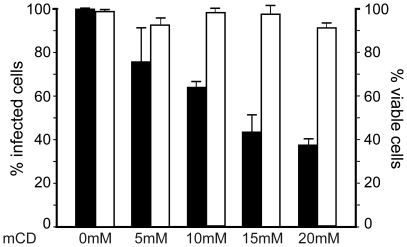
Methyl β-cyclodextrin treatment decreases infection of mammalian cells by CHIKV. HEK293T cells were incubated in medium alone or medium supplemented with increasing concentrations of methyl β-cyclodextrin (mCD) and challenged with the 37997-GFP CHIKV strain (m.o.i. of 5). The percentage of GFP expressing cells was determined by flow cytometry analysis (black histograms). For each experiment, the number of viable cells was determined after exclusion coloration with Trypan Blue (white histograms). Values are expressed as a percentage of untreated conditions. Each value is the mean of 3 separate experiments performed in triplicate ± SD.

Studies of alphaviruses have identified lipids as possible cofactors for critical steps in infection. Mainly, cholesterol is required *in vivo* to permit fusion of alphaviruses with the endosome membrane of the host cell [43]. To evaluate the role of membrane cholesterol in CHIKV infection, HEK293T cells were pre-treated with increasing concentrations of methyl β-cyclodextrin (mCD), a potent cholesterol depleting agent, and then challenged with the 37997-GFP CHIKV strain. Quantitative analysis of CHIKV infection using flow cytometry revealed that the percentage of CHIKV-positive cells was reduced by 63% at the highest dose of mCD used (20 mM) (Figure 3). This concentration of mCD was previously reported to significantly deplete membrane cholesterol and inhibit transferrin endocytosis [44]. Cell viability was unaffected by any dose tested in these experiments. Accordingly, mCD treatment significantly reduces CHIKV infection of HEK293T mammalian cells.

### Functional Rab5, but not Rab7, is required for infection of mammalian cells by CHIKV

**Figure 4:**
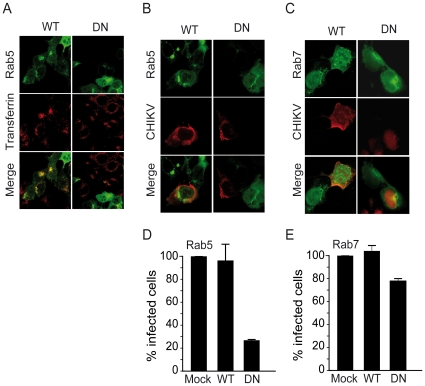
Role of Rab5 and Rab7 GTPases in CHIKV infection of HEK293T cells. HEK293T cells grown on coverslips were transfected with plasmids encoding either wild-type (WT) or dominant negative (DN) forms of GFP-tagged Rab5 (A), (B) and (D) or GFP-tagged Rab7 (C) and (E). Twenty-four hours post-transfection, cells were challenged with CHIKV (37997 strain used at a m.o.i. of 5). After another 4 hours in culture, the cells were permeabilized and viral antigens were labelled using anti-capsid C42 mAb and Alexa 594 secondary anti-Ig reagents. Antigen-expressing cells were revealed by confocal imaging (B) and (C), or quantified by flow cytometry (D) and (E). In control experiments, cells expressing GFP-fused WT or DN Rab5 were analyzed for Alexa Fluor 594-labelled transferrin uptake by confocal imaging (A). All experiments were repeated three times with similar outcomes. Images are representative of individual cells visualized and percentages of CHIKV positive cells determined by flow cytometry analysis are the mean of 4 separate experiments performed in triplicate ± SD.

To gain insight into the route of endosomal delivery of CHIKV into mammalian cells, HEK293T cells were transfected with GFP-tagged constructs expressing WT or DN forms of Rab5 or Rab7, two Rab GTPases required for transport of cargoes to early and to late endosomal vesicles, respectively. Confocal microscopy analysis revealed that disrupting Rab5 function by expression of a GFP-tagged DN form of this protein, inhibited transferrin uptake (Figure 4A). Then, transfected cells were challenged with the 37997 strain and processed for immunofluorescence. Intracellular CHIKV, detected with mAbs reacting with capsid antigen, was observed in cells overexpressing WT Rab5. In contrast, CHIKV antigens were rarely detected in cells expressing the DN Rab5 GTPase (Figure 4B). Similar experiments were performed using cells expressing a GFP-tagged WT or DN Rab7 transgene. After CHIKV challenge, the percentage of virus-positive cells was only slightly decreased by expression of DN Rab7 and was almost unaffected by over expression of WT Rab7 (Figure 4C). For quantitative studies, the fraction of infected cells was determined by flow cytometry analysis of CHIKV antigen-positive cells. As shown in Figure 4D, the expression of a DN Rab5 reduced by over 70%, the percentage of CHIKV-positive cells. In cells expressing the DN Rab7 transgene, CHIKV infection was only moderately reduced with a 30% decrease, when compared with cells expressing WT Rab7 (Figure 4E). Accordingly, entry of CHIKV in mammalian cells requires the integrity of early endosomes whilst disruption of the late endosomal compartment has only a very moderate impact on CHIKV infection.

### Role of the cytoskeleton network in CHIKV endocytosis

**Figure 5:**
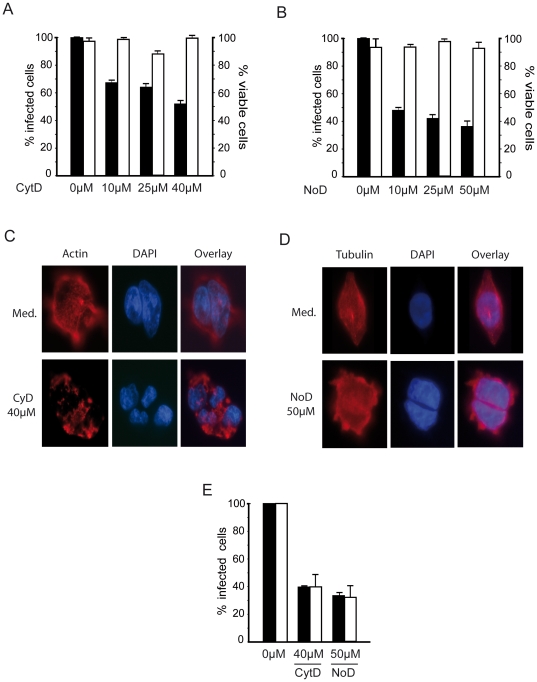
Effect of actin-disrupting drug and microtubule depolymerizating agent on CHIKV infection. HEK293T cells were treated with increasing concentrations of (A) cytochalasin D (Cyt D) or (B) nocodazole (NoD) before exposure to the 37997-GFP strain of CHIKV (m.o.i. of 5) (black bars). Sixteen hours post-infection, the percentage of infected cells was determined by quantification of GFP-expressing cells by flow cytometry analysis. For each point, cell viability was determined by Trypan blue exclusion and expressed as a percentage of untreated controls (white bars). Values are the mean of 3 separate experiments performed in duplicate + SD. Organization of cytoskeleton in cells cultured in medium alone (Med) or supplemented with cytochalsin D (C) or nocodazole (D) was analyzed by immunofluorescence after labelling with anti-actin (C) or anti-tubulin mAbs (D). Nuclei are stained with DAPI. (E) Flow cytometry analysis of CHIKV infected cells after treatment with cytochalasine D or nocodazole and viral challenge performed in neutral (black bars) or acid (white bars) medium. Each value is the mean of 3 separate experiments performed in duplicate ± SD.

Microtubules and actin microfilaments form most of the cytoskeletal framework of the cell that determine, with the help of motor proteins, endosomes and proteins and cargoes trafficking within the cells [45], [46], [47]. Since the endosomal compartment is required for CHIKV infection, the participation of the cytoskeleton can be inferred. In order to examine the role of intact cytoskeleton microfilaments during infection, cells were treated with specific depolymerizating agents prior to infection with CHIKV. First, actin destabilization was achieved with cytochalasin D, a fungal compound that disrupts microfilaments by binding to the fast-growing end of the actin fiber [48]. The capacity of drug treatment to destabilize actin fibers in HEK293T cells was ascertained by immunofluorescence staining of actin (Figure 5C). Then the cells were challenged with 37997-GFP CHIKV. When compared with untreated cells, actin fiber disruption reduced the percentage of infected cells by 48% without decreasing cell viability (Figure 5A). The structural integrity of the actin cytoskeleton is thus required for CHIKV infection. We next examined the role of microtubules in CHIKV infection using nocodazole, a reversible mitotic inhibitor that binds to β-tubulin and interferes with microtubule polymerization (Figure 5D). When cells treated with increasing concentrations of this drug, ranging from 10 µM to 50 µM, were challenged with 37997-GFP CHIKV, the percentage of GFP-expressing cells was found reduced from 52% to 68% when compared with cells maintained in medium supplemented with drug solvent (Figure 5B). Cell viability was not significantly affected as assessed by Trypan blue exclusion analysis. To discriminate between the effects of inhibitors on entry or downstream processes, like gene expression, we performed acid mediated by-pass assays. CHIKV particles were bound to cells pretreated with cytoskeleton depolymerizating agents. Cells were maintained at 4°C, and then, medium was acidified to pH 5.0 to stimulate viral fusion. After washes, infection was allowed to proceed for 16 hours before analysis quantification of CHIKV-positive cells by flow cytometry. As shown in Figure 5E, cytochalasine D or nocodazole reduced infection levels in similar extent when cells were challenged with CHIKV in neutral or acidic medium. Accordingly, medium acidification did not by-passed blockade in CHIKV replication caused by cytoskeleton disruption. Thus, microtubules and actin-fibers integrity is required, at least, for a post-fusion step of CHIKV life cycle.

### Comparative study of entry pathways recruited by African and Indian Ocean strains of CHIKV

**Figure 6:**
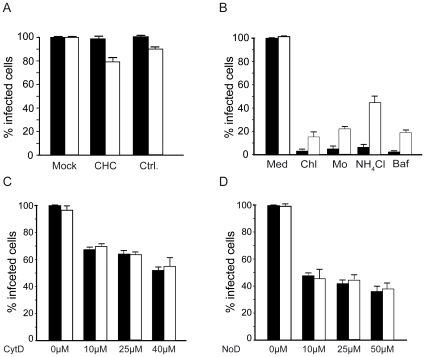
Comparative analysis of entry pathways used by the 37997 African strain and the LR-OPY1 isolate to infect HEK293T cells. Cells were challenged with normalized amounts (m.o.i. of 5) of the 37997-GFP (black bars) or the LR-OPY1-GFP (white bars) strains of CHIKV after (A) transfection of cells with siRNA directed to clathrin heavy chain (CHC) or control siRNA (Ctrl), or after treatment with (B) lysomotropic agents (chloroquine (Chl), monensin (Mo), amonium chloride (NH_4_Cl) or bafilomycin A1 (Baf)) or (C) cytochalasine D (CytD) or (D) nocodazole (NoD) in conditions described inMaterial and Methods. For each experiment, a control performed by incubating the cells in the presence of medium supplemented with an equivalent amount of drug solvent (Med) is included. Twenty-four hours post-infection, CHIKV replication was monitored by flow cytometry analysis of GFP expression. Each value is the mean of 3 separate experiments performed in triplicate ± SD.

Strains circulating recently in the Indian Ocean have been phylogenetically related to the African prototype of CHIKV. A main divergence has been reported in the presence of an alanine-to-valine mutation at position 226 in the E1 gene [26]. This mutation has been related to the high pathogenicity observed in some patients during the Indian Ocean outbreak. Moreover, E1-A226V was found to improve CHIKV dissemination in *Aedes albopictus* mosquitoes and increases dependence on cholesterol for *in vitro* replication in mosquito cell lines [30]. Entry pathways recruited by the LR-OPY1-GFP isolate to infect mammalian cells were thus investigated and compared with data obtained in the present study using the 37997-GFP African strain (Figure 6). Overall both strains were found to display a similar sensitivity regarding expression of an Eps15 dominant negative transgene (data not shown) and knock-down of clathrin heavy chain (Figure 6A). The main difference observed when both CHIKV isolates were compared was evidenced in infection assays performed using cells treated with lysomotropic agents as a target (Figure 6B). Interestingly, despite sensitive to pH acidification inhibitors, the LR-OPY1-GFP isolate was 4 to 5 times less sensitive to NH_4_Cl treatment than the 37997 African strain. Considering chloroquine, monensin and bafilomycin A1, this tendency was confirmed with a significantly reduced sensitivity of LR-OPY1 CHIKV to these chemical (p<0.031 in any case). Finally, considering host cytoskeleton disrupting agents, sensitivity to cytochalasin D and nocodazole was similar for the LR-OPY1 and the 37997 strain of CHIKV (Figure 6C and 6D). These data suggest that pathways used by the 3997-GFP African strain and the LR-OPY1-GFP isolate may differ in some extent.

## Discussion

The general entry model of alphaviruses into target cells relies on endocytosis in the cell cytoplasm of receptor-bound viral particles before the viruses are routed through endosomes along the endocytic trafficking pathway. Fusion of the endosome and virus membranes and entry of the viral capsid into the host cell cytoplasm then requires the acidification of the environmental pH [49], [50], [51]. Despite this general picture, a number of studies evidenced marked differences with respect to entry mechanisms used by closely related viruses, even when clustered within the closely related subgroup of New World or Old World alphaviruses. For example SFV, the prototype Old World alphavirus enters the target cell by a the clathrin-dependent endocytosis and pH-acidification [32] whereas entry of another Old World virus, SINV may, according to some studies, not require the acidification of the endocytic compartment [33]. Post-internalization, viruses may follow two different endocytic trafficking routes: 1) the recycling endosomes route or 2) the lysosome-targeted pathway where acidification occurs and facilitates uncoating and fusion of enveloped viruses. In the lysosomal pathway, the nature of endosomes supporting the fusion events has been a matter of debate. Several viruses from the alphavirus genus (e.g. SFV) require Rab5-positive early endosomes but not a functional Rab7-positive late endosomal compartment for productive entry and infection [52], [53]. Yet, other members of the alphavirus genus (e.g. Venezuelan encephalitis virus) need to migrate through both early and late endosomes before fusion can occur and viral genome is released into the cytoplasm [34]. According to these divergences, the routes utilized by CHIKV to infect its target cells cannot be inferred based on previous experiments performed from related viruses such as SFV and SINV. In this report we used diverse chemical and molecular inhibitors together with dominant negative proteins to explore CHIKV entry in mammalian cells. We demonstrated that CHIKV entry into epithelial HEK293T cells requires the presence, in the target cell, of functional Eps15. Eps15 specifically contributes to the severing of clathrin-coated vesicles, but also performs an important function in coupling ubiquitinated cargo to clathrin-independent internalization [41]. Knock-down of clathrin-heavy chain helped deciphering with those internalization pathways and showed that CHIKV infection is independent of clathrin-coated vesicles formation. This result was reproduced using the Hela cell line, a second model of epithelial cells. These observations indicate that entry of CHIKV into epithelial cells can be mediated, at least in some cell types, by a clathrin-independent endocytosis.

The endosomal compartment required for CHIKV entry was investigated by mean of overexpression of dominant negative mutants of Rab-family GTPases that regulate transport, sorting and maintenance of endosomal vesicles along the endocytic pathway. Rab5 associates to early endosomal vesicles and is required for the transport of early endosomes along the microtubules [46], [54]. Rab7 is a marker for late endosomes and its activity regulates sorting of cargoes from early endosomes toward the late endosome/lysosome pathway [55]. Overexpression of DN Rab5 and DN Rab7 mutants that result in constitutive inactivation of endosome biogenesis has been widely used to study trafficking pathways of viruses within the vesicular compartment [55], [56]. Here, we found that overexpression of DN Rab5 reduces the susceptibility of mammalian cells to CHIKV infection, supporting the hypothesis that this virus must access early endosomes in order to productively infect mammalian cells. In respect to this finding, CHIKV is similar to SFV that requires a functional Rab5 for entering into its target [52]. In contrast, the requirement for a functional Rab7-positive compartment is less clear since only a moderate inhibition of CHIKV infection was observed using DN Rab7 transfected cells. The role of Rab7 in CHIKV entry needs further investigations. Indeed, despite dispensable for SFV fusion which occurs in Rab5-positive endosomes, enrichment with Rab7 and subsequent migration of the virus contained in these Rab7-enriched domains along microtubules was reported for SFV [52].

Once within the endosomal compartment, fusion of alphaviruses with the host cell membrane depends upon a low pH microenvironment. For SFV, acidification allows insertion of viral spike glycoproteins into the endosomal membrane and subsequent delivery of the nucleocapsid into the cytoplasm [32]. All lysomotropic agents tested in the present study resulted in the drastic inhibition of CHIKV infection, supporting the hypothesis that entry in mammalian cells is strictly dependent upon pH-acidification of intracellular vesicles. Analyzing the consequences of cytoskeleton disruption using polymerization inhibitors, we found that CHIKV infection requires both intact actin microfilaments and microtubules. The contribution of actin underlying the plasma membrane to endocytosis [57], [58], and also the function of microtubules in endosomal traffic from peripheral early to late endosomes [57] render these microfilament networks essential not only for initial internalization of cargoes and viruses, but also for transportation through endocytosis vesicles and finally in allowing viruses to reach the nuclear compartment [59], [60], [61]. Having demonstrated here that a productive CHIKV infection requires a functional early endosomal compartment, the decreased viral infection observed following actin and microtubule disruption may thus reflect the requirement of intact cytoskeleton for endosomal trafficking. Interestingly, the inability of acid mediated by-pass to restore infectivity in cells treated with cytoskeleton depolymerizating agents suggests that integrity of actin fibers and microtubules is not only required for viral internalization, but also for a post-fusion step of the viral life cycle.

Together with previously published informations [40], our data indicate that CHIKV enters epithelial mammalian cells through a clathrin-independent, Esp15-dependent, dynamin 2-dependent route, requires a functional early endosomal compartment and is dependent on endosomal acidification to reach productive infection. Further studies will help to identify precisely the nature of this entry pathway. It has to be kept in mind that virus penetration in the target cell generally results from a balanced usurpation of multiple endocytic pathways. In the present study, to the exception of lysomotropic agents that drastically inhibited CHIKV infection, viral entry was in any case only partly inhibited by chemical drugs or dominant negative molecules interfering with the cell endocytosis. This may support the hypothesis that several pathways are hijacked by CHIKV to penetrate into its target cells. In addition, entry pathways hijacked by viruses may vary according to the target cell type [62]. In support of this idea, we recently found that CHIKV productively infects the human hepatoma HuH7 cell line [35] devoid of cav-1 required for caveolar vesicles formation [63].

Overall entry pathways are mostly shared by the 37997 African strain and the LR-OPY1 Reunion Island isolate of CHIKV analyzed in the present study. Both viruses were equally impaired by inhibition of Eps15 and cytoskeleton disruption. Although the amino-acid residue present at position 226 within the E1 envelope ectodomain was previously proposed to regulate the interaction of the fusion loop with cholesterol in the target cell membrane [29], [64], we found that infection by the 37997 African prototype of CHIKV (bearing a A226 a.a.) or by the LR-OPY1 isolate (bearing a V226 a.a.) are equally inhibited by cholesterol-depleting agents. These data contrast with other observations demonstrating that the presence of the A226V mutation in the E1 gene is related to different requirement for membrane cholesterol to infect mosquito cell lines *in vitro* [30]. The main difference observed between both strains is that LR-OPY1 strain is less dependent on pH acidification of endosomes to infect mammalian cells. Requirement for low endocytic pH observed for CHIKV in the present study is shared with SFV [49], [50] but diverges from that of SINV, another Old World alphavirus that can, in some cases establish productive infection without the need for a pH-dependent endocytic pathway [33], [65]. Although the molecular mechanisms underlying such differences are likely complex and elusive, it is tempting to speculate that the genetic drift of viral isolates circulating in the Indian Ocean area during the recent epidemic [26] may be associated with the acquisition of alternative entry mechanisms. Such an evolution has to be considered in developing new antiviral strategies in response to a potential worldwide spread of the Chikungunya virus.

## Supporting Information

Figure S1 Role of clathrin-dependent endocytosis in CHIKV infection of Hela cells. Subconfluent cultures of Hela cells were left unstransfected (Mock) or transfected with siRNA targeting the clathrin heavy chain (CHC) or with control siRNA (Ctrl.). (A) Level of CHC expression in transfected cells was analyzed by immunoblot. Actin expression was used to monitor proteins level in each sample. (B) Transferrin uptake control was performed by incubating cells with Alexa-594-labelled transferrin (5 µg/ml) for 30 min at 37°C. After washes, cells were fixed and processed for fluorescence imaging. (C) 72 h post-transfection, the cells were challenged with CHIKV (m.o.i. of 5). After another 16 h in culture, cells were fixed and permeabilized, and viral antigens were labelled with mAbs C42 and Alexa Fluor 594-conjugated anti-mouse Ig secondary antibody. Percentage of infected cells was determined by flow cytometry. Values are the mean of three independent performed in duplicate ± SD.
(1.82 MB TIF)

Figure S2 Characterization of 37997-GFP and LR-OPY1-GFP CHIKV strains replication in HEK293T cells. Replication kinetics of increasing concentrations of (A) 37997-GFP CHIKV or (B) LR-OPY1-GFP CHIKV in HEK293T cells was established flow cytometry analysis of GFP expression at 7 h, 14 h and 24 h post-infection (p.i.). Data are representative of 5 separate experiments. It is worth noting that CHIKV is highly cytopathic and infected cell cultures cannot be maintained for long periods of time.
(1.32 MB TIF)
