# Supplementary figures and images for: Endocytosis of Chikungunya Virus into Mammalian Cells: Role of Clathrin and Early Endosomal Compartments
Source: PLoS One. 2010 Jul 8;5(7):e11479. doi: 10.1371/journal.pone.0011479 (PMC2900206; doi:10.1371/journal.pone.0011479)

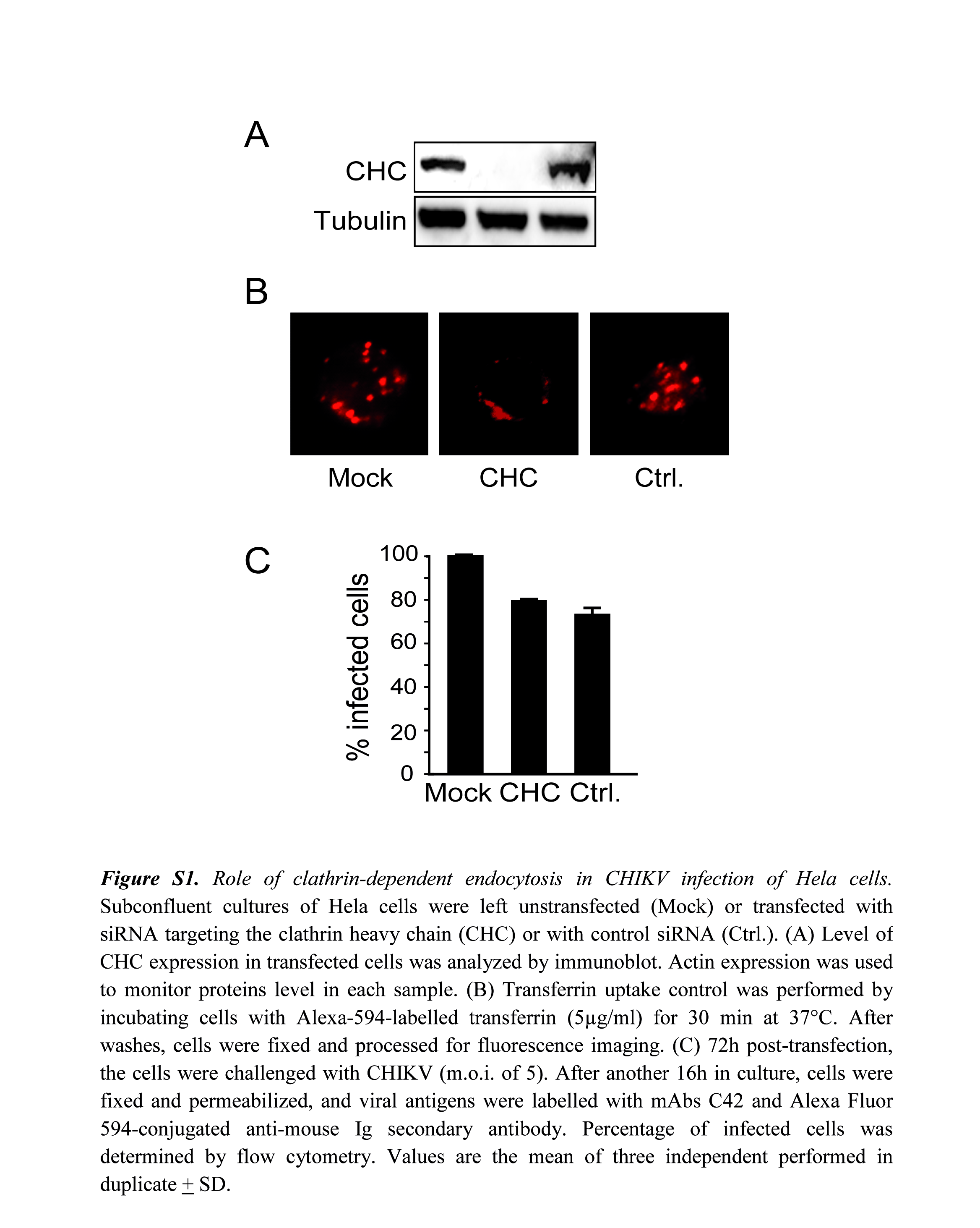

Supplement: Figure S1 — Role of clathrin-dependent endocytosis in CHIKV infection of Hela cells. Subconfluent cultures of Hela cells were left unstransfected (Mock) or transfected with siRNA targeting the clathrin heavy chain (CHC) or with control siRNA (Ctrl.). (A) Level of CHC expression in transfected cells was analyzed by immunoblot. Actin expression was used to monitor proteins level in each sample. (B) Transferrin uptake control was performed by incubating cells with Alexa-594-labelled transferrin (5 µg/ml) for 30 min at 37°C. After washes, cells were fixed and processed for fluorescence imaging. (C) 72 h post-transfection, the cells were challenged with CHIKV (m.o.i. of 5). After another 16 h in culture, cells were fixed and permeabilized, and viral antigens were labelled with mAbs C42 and Alexa Fluor 594-conjugated anti-mouse Ig secondary antibody. Percentage of infected cells was determined by flow cytometry. Values are the mean of three independent performed in duplicate ± SD. (1.82 MB TIF) [file pone.0011479.s001.tif]

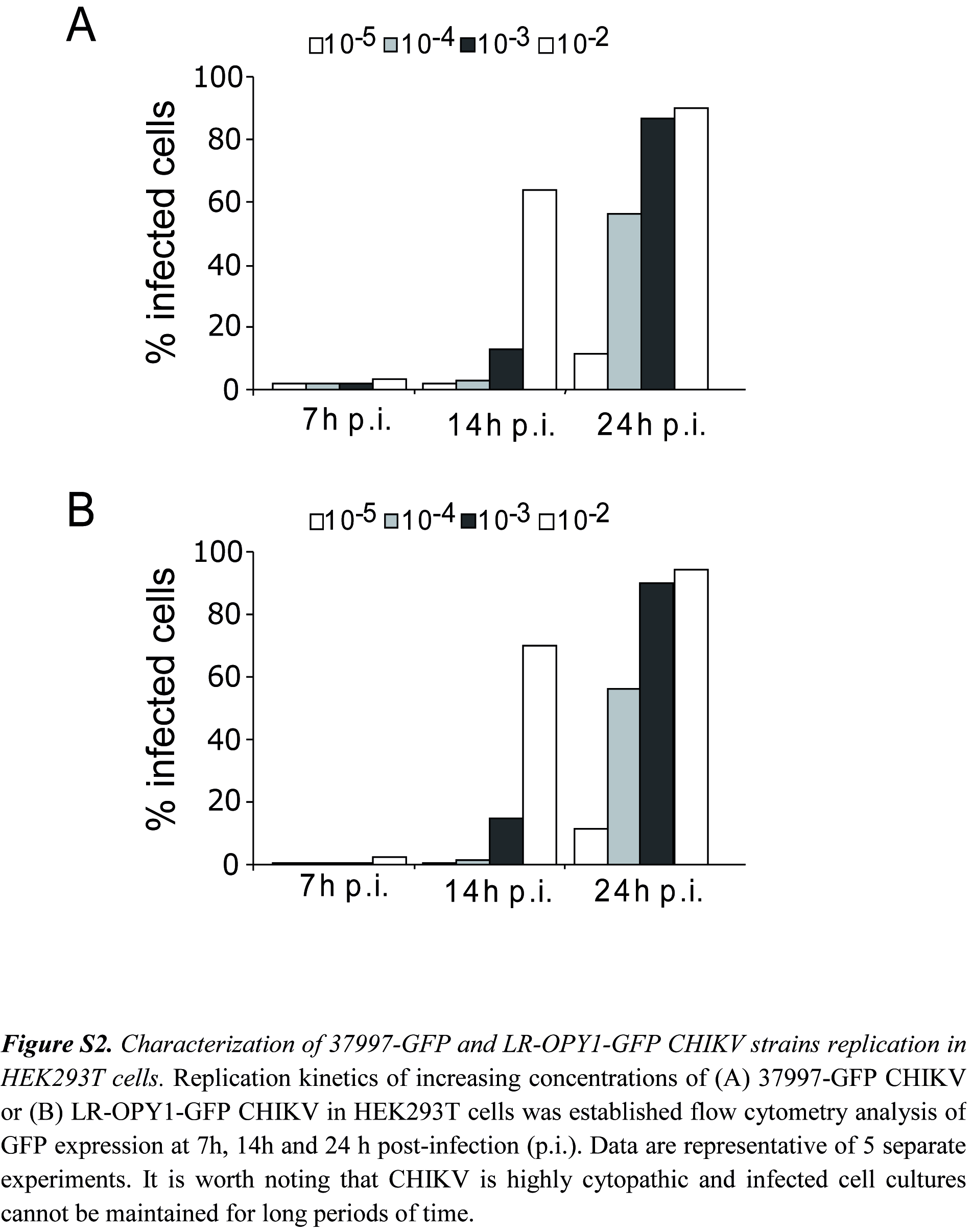

Supplement: Figure S2 — Characterization of 37997-GFP and LR-OPY1-GFP CHIKV strains replication in HEK293T cells. Replication kinetics of increasing concentrations of (A) 37997-GFP CHIKV or (B) LR-OPY1-GFP CHIKV in HEK293T cells was established flow cytometry analysis of GFP expression at 7 h, 14 h and 24 h post-infection (p.i.). Data are representative of 5 separate experiments. It is worth noting that CHIKV is highly cytopathic and infected cell cultures cannot be maintained for long periods of time. (1.32 MB TIF) [file pone.0011479.s002.tif]
